# Modulating Membrane Surface Properties via Prewetting With Polysorbate 20 to Improve Sterile Filtration of Nanoemulsions

**DOI:** 10.1002/biot.70280

**Published:** 2026-07-09

**Authors:** Shreya Kapila, Milea M. Osmanski, Randal J. Soukup, Marissa E. Bradley, David Boyd, Andrew L. Zydney

**Affiliations:** ^1^ Department of Chemical Engineering The Pennsylvania State University University Park Pennsylvania USA; ^2^ Merck & Co., Inc. Rahway New Jersey USA

**Keywords:** bioprocessing, membrane fouling, nanoemulsions, polysorbate, sterile filtration, surfactant

## Abstract

Nanoemulsions are promising vaccine adjuvants and drug delivery vehicles. However, sterile filtration of nanoemulsion formulations remains challenging because of the large size of the nanodroplets. This study investigates how prewetting the sterile filters with Polysorbate 20 (PS20) modulates the surface properties and enhances filtration performance of a range of sterile filters using a squalene‐based nanoemulsion stabilized with PS20 and Span 85. FTIR and contact angle measurements were used to evaluate the change in filter surface chemistry. Prewetting with PS20 reduced the buffer permeability, but it generally increased the filtrate flux with the nanoemulsion, often by as much as a factor of two, due to the reduction in frictional interactions between the surfactant‐coated nanodroplets and the pretreated membrane. PS20 also reduced the extent of pore blockage, leading to a dramatic increase in capacity with most sterile filters. For example, the capacity of the Millipore Express Plus increased by nearly 100‐fold, compared to only a 2‐fold increase in capacity for the dual‐layer Pall Supor 0.8/0.2 µm filter, even though both filters are polyethersulfone. This work highlights the importance of membrane surface chemistry and the potential for modulating filter properties by prewetting with surfactants to improve the sterile filtration of nanoemulsion‐based drug products.

## Graphical Abstract and Lay Summary

Nanoemulsions are promising vaccine adjuvants and drug delivery vehicles. In this work, we have demonstrated that sterile filtration performance can be significantly enhanced by prewetting the sterile filter with Polysorbate 20 to increase the hydrophilicity of the sterile filter.

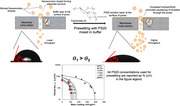

## 1 Introduction

Nanoemulsions (NEs) composed of immiscible oil and aqueous phases, stabilized using appropriate surfactants, have emerged as potent vaccine adjuvants capable of enhancing immunogenicity through improved antigen uptake, presentation, and activation of the innate immune response [1, 2]. Well‐established systems such as MF59 (squalene‐based oil‐in‐water emulsion adjuvant) and AS03 (Adjuvant System 03, a squalene‐based oil‐in‐water emulsion containing α‐tocopherol) have demonstrated robust and durable immune stimulation, highlighting the promise of NE‐based adjuvants for next‐generation vaccines with optimized safety and efficacy [3, 4, 5, 6]. NEs can also be used as delivery vehicles for poorly soluble hydrophobic drugs, significantly increasing bioavailability.

Sterile filtration is a critical step in the downstream processing of injectable NE formulations, ensuring removal of all microorganisms without compromising the quality of the formulated drug product. However, several recent studies have shown that NEs cause rapid fouling of sterile filters, leading to low capacities and higher costs [7, 8, 9]. The development of strategies to mitigate the extent of fouling is critical to improve process robustness and product consistency.

Polyoxyethylene (20) sorbitan monolaurate (Polysorbate 20, often referred to as Tween 20) is a widely used nonionic surfactant commonly incorporated in parenteral formulations to stabilize both oil‐in‐water NEs and protein‐based products [10, 11]. PS20 readily adsorbs to both hydrophilic and hydrophobic polymeric membrane materials [12], including polyvinylidene fluoride (PVDF) and polyethersulfone (PES), forming a surface layer that modifies interfacial properties. Ren et al. [13] showed that PS20 adsorption ranged from 41 µg/cm^2^ on a hydrophilized PES membrane to more than 500 µg/cm^2^ on a mixed cellulose ester membrane. Zhang et al. [14] used neutron reflectometry to study PS20 adsorption to polystyrene surfaces, with the results consistent with the formation of a 2.1 nm film on the polymer. Mahler et al. [15] showed that PS20 transport through ultrafiltration membranes was highly variable (and non‐predictable), likely due to differences in interactions with the membrane, while there was minimal loss in PS20 during filtration through larger pore size sterile filters.

Previous studies have also demonstrated potential opportunities to use surfactant or polymer coatings to improve membrane hydrophilicity and antifouling performance. Xie et al. [16] showed that it was possible to modify polypropylene membranes by adsorption of various polysorbate surfactants (Polysorbate 20, 40, 60, 80, and 85). The membranes treated with polysorbate were more hydrophilic and under some conditions showed higher water permeability than the base polypropylene membrane. Boributh et al. [17] and Zhang et al. [18] demonstrated that modifying PVDF membranes with chitosan or tannin coatings, respectively, significantly increased membrane hydrophilicity and reduced the extent of fouling.

Our recent work on sterile filtration of NEs [8, 9] demonstrated that there was negligible flux until the transmembrane pressure (TMP) exceeded a critical value required to force the nanodroplets into and through the membrane pores, suggesting that both the membrane pore size and surface properties (e.g., hydrophilicity) might play an important role in governing the filtration behavior. The objective of this work was to specifically examine the effects of prewetting with different concentrations of PS20 on the surface properties and filtration performance of several different sterilizing‐grade filters. The extent of PS20 deposition was examined using a combination of FTIR, contact angle, and permeability measurements. The effects on the sterile filtration behavior were evaluated by performing both constant pressure and constant flux filtration experiments using a model NE. This integrated approach provides important mechanistic insights into the role of surfactant–membrane interactions on the sterile filtration performance while highlighting the potential of using PS20 to enhance the performance during sterile filtration of NE‐based drug products.

## 2 Materials and Methods

### 2.1 Nanoemulsions

Oil‐in‐water NEs were formulated following the method previously described by Kapila et al. [8]. Briefly, squalene, PS20 (polyoxyethylene sorbitan monolaurate), and Span 85 (sorbitan trioleate) were combined with 20 mM L‐histidine buffer at pH 5.8 (all reagents from MilliporeSigma, St. Louis, MO). This mixture was initially homogenized using a Silverson L5MA mixer (Silverson Machines, Inc., East Longmeadow, MA) to create a coarse emulsion with droplet size near 1 µm as measured by dynamic light scattering (DLS). This emulsion was subsequently subjected to high‐pressure homogenization using a GEA Panda Plus 2000 unit (GEA Group, Parma, Italy), operating at 140,000 kPa (20,000 psi) for 10 passes, resulting in a NE with an average droplet diameter of approximately 160 nm [8]. The resulting NE was stored at 4°C and equilibrated to room temperature (21°C ± 2°C) immediately prior to use in the filtration experiments. NE concentrations were determined by measuring the absorbance at 450 nm using a Tecan microplate reader (Mannedorf, Switzerland) in 96‐well clear plates; the absorbance is due to contributions from both the squalene and surfactants.

### 2.2 Sterile Filtration

Sterile filtration experiments were performed using sterile filters having different pore morphology and surface chemistry as summarized in Table 1. The hydrophilic version of the Durapore PVDF membrane has an acrylate modification to increase the wettability [17]. Filters were used either in syringes, capsules, or as discs, with the latter placed in a stainless‐steel holder and sealed with an O‐ring. Sterile filtration was performed at both constant flux and constant pressure. A constant filtrate flux of 42 L/m^2^/h was maintained using a Masterflex peristaltic pump positioned immediately upstream of the sterile filter, with the permeate outlet open to the atmosphere. The upstream pressure was monitored using a Track‐it pressure gauge (Monarch Instrument), with data logged every second using Track‐It Logger Software. Constant pressure filtration experiments were performed at 280 kPa (40 psi) based on our previous work [8]. The applied pressure was set by air pressurization of the feed reservoir, with the pressure monitored using an Ashcroft digital pressure gauge (Stratford, CT). The cumulative filtrate volume was evaluated by timed collection, with the permeate mass measured by an OHAUS Ranger 3000 analytical balance using the OHAUS Serial Port Data Collection Software.

**TABLE 1:** Summary of sterile filters examined in this work. Hydraulic permeabilities after PS20 were obtained by prewetting the sterile filter with a solution containing 20% PS20 in histidine buffer. Mean permeability and standard deviation were evaluated from data obtained with three separate membranes for each filter type.

Sterile filter	Morphology	Pore size (µm)	Chemistry	Module	Hydraulic permeability (L m^−2^ h^−1^ kPa^−1^)	Hydraulic permeability after PS20 (L m^−2^ h^−1^ kPa^−1^)
Pall Supor	Dual layer, asymmetric	0.8 | 0.2	PES	Syringe	150 ± 70	97 ± 15
Pall Supor EKV		0.65 | 0.2	PES	Disc	120 ± 40	67 ± 2
Sartopore 2 XLG		0.8 | 0.2	PES	Capsule	102 ± 15	96 ± 21
Millipore Express Plus	Asymmetric	0.22	PES	Disc	134 ± 3	115 ± 9
Pall Supor		0.2	PES	Disc	93 ± 6	35 ± 9
Durapore	Homogeneous	0.22	PVDF (hydrophilic)	Disc	60 ± 3	52 ± 2
Durapore		0.22	PVDF (hydrophobic)	Disc	—^a^	61 ± 5
Isopore		0.2	Polycarbonate track‐etched	Disc	45 ± 13	38 ± 5

^a^ Permeability with water could not be measured because the membrane is too hydrophobic to wet with water alone.

The manufacturers typically recommend wetting the sterile filters with water or buffer while venting the capsule to eliminate any entrapped air. In this work, prewetting was performed using 20 mM L‐histidine buffer with different amounts of added PS20; the resulting solutions were stirred at 450 rpm for 5 min using a magnetic stirrer to ensure good dispersion of the surfactant before prewetting. Approximately 50 L/m^2^ of the buffer + PS20 solution was filtered through the sterile filter at a flux of 1500 L/m^2^/h followed by 50 L/m^2^ of the 20 mM histidine buffer (without PS20) to remove any free surfactant from the system. The feed reservoir was then replaced with the NE to start the constant flux filtration experiment.

### 2.3 Filter Characterization

The hydraulic permeability (*L*_*p*_) of the sterile filters was evaluated using 20 mM L‐histidine buffer by measuring the TMP over a range of filtrate flux (*J*_*v*_):

(1) $$L_{p}=\frac{J_{v}}{\mathrm{TMP}}$$

Initial permeability values (Table 1) were evaluated using buffer alone, with the permeability for the same membrane evaluated after prewetting with a 20% PS20 solution (without air drying). The 20% *v*/*v* PS20 solution was prepared by mixing 2.5 mL of PS20 in 10 mL of histidine buffer, yielding a final volume of 12.5 mL. All PS20 concentrations are expressed as % *v*/*v* throughout the article.

Fourier transform infrared (FTIR) spectroscopy was performed to evaluate potential changes in the membrane surface properties after prewetting with PS20. FTIR spectra were obtained using a Vertex 70 spectrometer (Bruker Optics, Billerica, MA) equipped with a mercury cadmium telluride (MCT) detector cooled by liquid nitrogen. Membranes were examined after prewetting with buffer or PS20, following the procedures described above, and subsequently air‐dried for 48 h to remove water. Measurements were performed in attenuated total reflection (ATR) mode using a DiaMax single‐bounce diamond ATR accessory (Harrick Scientific, Pleasantville, NY) set at a 45° incident angle. Each spectrum was recorded by averaging 400 scans at a resolution of 4 cm^−^^1^. Absorbance spectra were baseline‐corrected by referencing to a clean, unused ATR crystal. Between samples, the crystal was cleaned with 2‐butanone; crystal cleanliness was verified by spectral analysis after cleaning.

Contact angle measurements were conducted to assess changes in membrane hydrophilicity following PS20 prewetting using a Ramé–Hart Model 260 automated goniometer (Succasunna, NJ) for membranes prewet with buffer or buffer + PS20 and then air‐dried for 48 h. A 10 µL droplet of deionized water was gently dispensed onto the membrane surface, with the droplet size, contact angle, and tilt captured within 0.15 s using DROPimage software. The contact angle for each membrane type was measured in triplicate (i.e., for three different membranes), with results presented as the mean ± the standard deviation.

Size exclusion chromatography (SEC) analysis was performed to quantify PS20 adsorption (or deposition) onto the membrane by measuring changes in PS20 concentration between the feed and permeate samples obtained during filtration of PS20 alone. PS20 was added to the buffer as described in Section 2.2, and the resulting solution was filtered through the membrane using the same procedures employed for the NE filtration experiments. SEC analysis was performed using an Agilent 1260 Infinity II High Pressure Liquid Chromatography (HPLC) system (Agilent Technologies, Santa Clara, CA) equipped with an Agilent Bio‐SEC column packed with 3 µm silica particles with a pore size of 100 Å. L‐histidine buffer (pH 5.8) was used as the mobile phase at a constant flow rate of 0.35 mL min^−^^1^. The column temperature was maintained at 25°C. PS20 was detected by UV absorbance at 260 nm using an Agilent 1260 Infinity variable wavelength detector. SEC peak areas were obtained by direct integration and used for relative comparison of PS20 content between the feed (before membrane filtration) and permeate samples.

Scanning electron microscopy (SEM) was used to examine visible changes in the surface pore morphology before and after prewetting (followed by a buffer flush). The sterile filter was carefully removed from the module and allowed to air dry in a fume hood at room temperature for 24 h. Dried membranes were then sectioned into smaller pieces, mounted on conductive stubs using double‐sided carbon tape, and coated with a thin layer of gold/platinum using a Bal‐tec SCD 050 sputter coater to minimize charging effects. SEM imaging was performed on a Zeiss SIGMA VP field emission scanning electron microscope (FESEM) operated at an accelerating voltage of 3.0 kV.

## 3 Results and Analysis

### 3.1 PS20 Adsorption/Deposition During Prewetting

**FIGURE 1:**
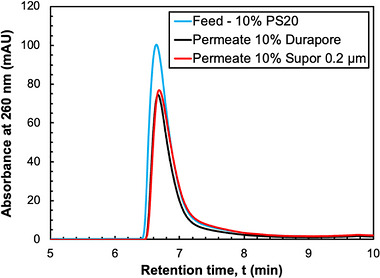
SEC chromatograms of the feed (blue) and permeate samples obtained during prewetting of the Durapore hydrophilic PVDF (black) and Supor 0.2 µm PES (red) sterile filters with 20 mM Histidine buffer containing 10% PS20.

The sterile filters used in this work were all prewet using either the 20 mM L‐histidine buffer alone or the same buffer but with different concentrations of PS20. Figure 1 shows representative SEC chromatograms of the feed and pooled permeate obtained during prewetting of the hydrophilic PVDF (Durapore) and PES (Supor 0.2 µm) sterile filters with a 10% PS20 solution. The 10% PS20 condition was selected for SEC analysis because it provided the most reliable quantification within the operating range of the SEC method without requiring sample dilution. The Durapore PVDF and Supor PES membranes were included as representative examples to compare surfactant adsorption behavior across different membrane materials. The permeate solutions show a small but significant reduction in peak intensity relative to the feed, providing direct evidence of PS20 deposition on and within the membrane during the prewetting. The PS20 concentration in the permeate solution is slightly higher for the 0.2 µm layer of the Supor PES filter, which would suggest a somewhat smaller amount of PS20 deposition compared to that for the Durapore. This is discussed in more detail subsequently.

**FIGURE 2:**
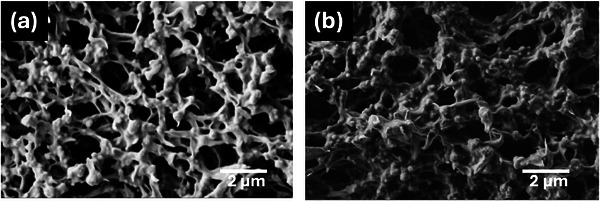
Scanning electron micrographs comparing the surface morphology of the hydrophilic Durapore 0.22 µm filter: (a) clean and (b) after prewetting with 20% PS20.

Prewetting with PS20 also caused visible changes in the surface of the filters. Figure 2 shows SEM images of the Durapore hydrophilic PVDF filter both clean (unused) and prewet with 20% PS20. The clean Durapore has an open pore structure with a sponge‐like, cross‐linked morphology. The filter prewet with PS20 shows a lower porosity, with many of the pores blocked or partially occluded by PS20 micelles (the critical micelle concentration for PS20 is only 0.005%–0.006% w/v [19], well below the concentrations used in these experiments). It was not possible to obtain similar images with the dual‐layer and asymmetric filters since the large majority of the PS20 deposition occurred within the depth of these structures, with minimal changes in the morphology of the external surface of the filter.

The effect of prewetting with a solution containing 20% PS20 on the permeability of the different sterile filters is shown in Table 1. Prewetting with PS20 reduced the buffer permeability of all the membranes, with the single exception of the Durapore hydrophobic PVDF for which it was not possible to evaluate the permeability of the pristine membrane due to its high hydrophobicity (there was no measurable flow through the filter using the histidine buffer up to a pressure of 280 kPa = 40 psi). In each case, the hydraulic permeability remained essentially constant after processing up to 36 L/m^2^ of histidine buffer through the prewet filters, suggesting that there was minimal removal of PS20 under these conditions. However, the magnitude of the reduction in permeability varied dramatically between the different sterile filters. Prewetting with 20% PS20 caused nearly a 3‐fold reduction in the permeability of the 0.2 µm layer of the Pall Supor membrane, from 93 ± 6 to 35 ± 9 L/m^2^/h/kPa (mean and standard deviation for data obtained with three separate membrane samples). This is considerably larger than the change in permeability for the Durapore hydrophilic PVDF even though the data in Figure 1 suggest that there is greater relative PS20 adsorption/deposition on the Pall Supor filter. One possible explanation for these differences may be related to the very different morphology of these membranes, with the Durapore having a relatively homogenous pore structure while the 0.2 µm layer of the Pall Supor filter is asymmetric (with the more open surface with larger pores facing the feed). No obvious correlation was observed between the change in permeability with either the initial permeability of the membrane (before prewetting) or the membrane morphology. For example, the dual‐layer Pall Supor EKV membrane showed nearly a 2‐fold reduction in permeability compared to the minimal change in permeability seen with the dual‐layer Sartopore 2 XLG even though both of these sterile filters are made of PES.

### 3.2 Surface Characteristics of Prewet Filters

**FIGURE 3:**
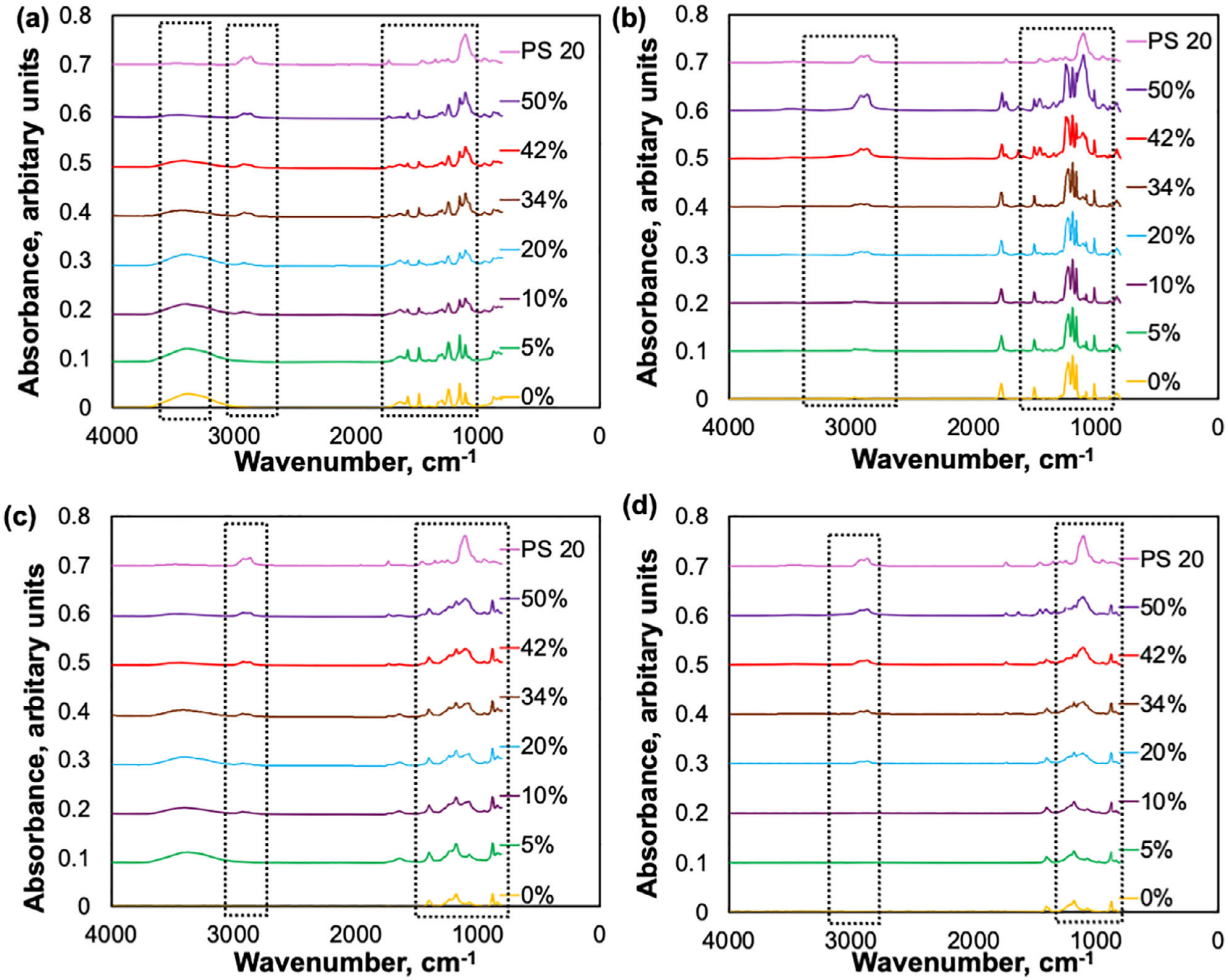
FTIR spectra of 0.2 µm single layer (a) Supor PES, (b) Isopore polycarbonate, (c) Durapore PVDF hydrophilic, and (d) Durapore PVDF hydrophobic membranes prewet with varying concentrations of PS20. FTIR spectrum for undiluted PS20 is shown for comparison. Spectra are offset vertically for clarity.

Additional insights into the effects of prewetting on the different sterile filters were obtained by FTIR spectroscopy. Figure 3 shows FTIR spectra for the 0.2 µm Supor PES, Isopore polycarbonate, Durapore PVDF hydrophobic, and Durapore PVDF hydrophilic membranes after prewetting with different concentrations of PS20, with the spectrum of undiluted PS20 included in each panel for reference. All spectra are vertically offset for clarity.

The FTIR spectrum of undiluted PS20 was obtained by analyzing the liquid sample directly, without deposition on a solid substrate. The spectrum exhibits several characteristic absorption bands associated with the polyoxyethylene sorbitan ester structure of PS20. Most notably, the distinct doublet in the 2850–2950 cm^−^^1^ region corresponds to symmetric and asymmetric C─H stretching vibrations of the aliphatic ─CH_2_ and ─CH_3_ groups. In addition, a strong band near 1735 cm^−^^1^ is assigned to ester C═O stretching, while bands in the 1100–1200 cm^−^^1^ region arise from C─O─C stretching vibrations of the polyoxyethylene chains. These PS20‐associated bands are also observed in the spectra for the membranes prewet with the PS20 solution. The intensity of the aliphatic C─H stretching bands near 2900 cm^−^^1^ and the ester C═O band near 1735 cm^−^^1^ increases with increasing PS20 concentration in the prewetting solution, consistent with a greater degree of PS20 adsorption/deposition on the membrane surface. Despite the presence of PS20, characteristic absorption bands of the underlying membrane polymers remain evident. For example, the Supor PES membrane shows peaks in the 1580–1600 cm^−^^1^ region attributed to aromatic ring C═C stretching, while strong absorptions near 1240–1320 cm^−^^1^ correspond to asymmetric stretching of the sulfone (─SO_2_─) groups characteristic of PES. The Isopore polycarbonate membrane exhibits a strong C═O stretching band near 1775 cm^−^^1^ and aromatic C═C stretching near 1505 cm^−^^1^, consistent with the carbonate linkage and phenyl rings of the polycarbonate backbone.

The Durapore PVDF membranes display characteristic C─F stretching and bending vibrations in the 1100–1400 cm^−^^1^ region, along with weaker C─H stretching features near 2900 cm^−^^1^ arising from the polymer backbone. These PVDF‐associated bands remain visible after wetting with PS20, indicating that the PS20 layer does not fully obscure the underlying membrane chemistry. Similar results were obtained by Wu et al. [20] after coating hollow fiber PVDF membranes with PS20 by simple adsorption (without filtration). Although it is difficult to quantify the extent of PS20 from the FTIR spectra, the peaks associated with PS20 are considerably more pronounced on the Isopore membrane (particularly the doublet near 2900 cm^−^^1^), although this could also reflect the much smoother surface of this track‐etched polycarbonate membrane. Interestingly, the Isopore membrane showed a relatively small reduction in permeability after prewetting with PS20 (from 45 ± 13 to 38 ± 5 L/m^2^/h/kPa), suggesting that much of the PS20 signal may be due to adsorption/deposition on the external polymer surface instead of within the membrane pores.

**FIGURE 4:**
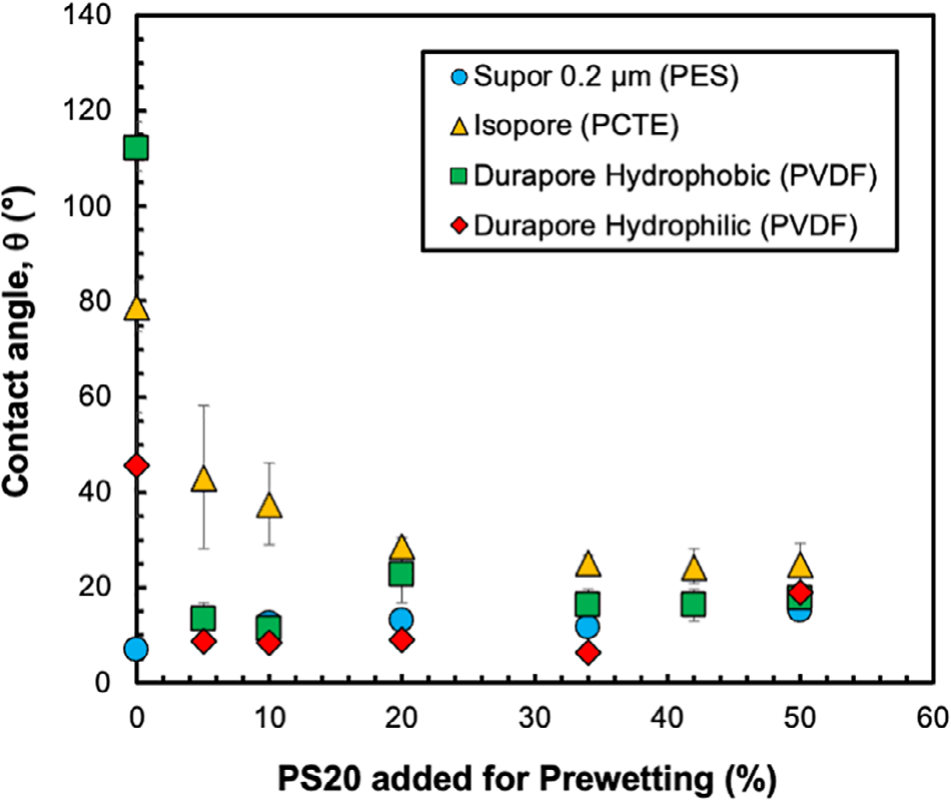
Water contact angles measured 1 s after depositing a water droplet on filters prewetted with different concentrations of PS20. Error bars represent the standard deviation for the contact angle evaluated with at least three different samples prewet under the same conditions.

Figure 4 shows the effects of prewetting with PS20 on the contact angle of the single layer Supor PES (0.2 µm layer), the 0.2 µm Isopore membrane, and the Durapore hydrophilic and hydrophobic PVDF sterile filters. The hydrophobic Durapore PVDF shows the largest initial contact angle when prewetted with buffer alone (112°), reflecting the intrinsic hydrophobicity of the base polymer. The Isopore polycarbonate membrane is moderately hydrophobic with an initial contact angle of ∼80°, while the Durapore hydrophilic (PVDF) and Supor 0.2 µm PES membranes display much lower contact angles (46 ± 11° and 7 ± 2°, respectively).

Prewetting with a solution containing even low concentrations (< 10%) of PS20 produces significant changes in the contact angle for all membranes tested, indicating strong surfactant–surface interactions even at low PS20 concentrations. The hydrophobic Durapore PVDF shows the largest absolute change, with the contact angle decreasing steeply from ∼112° (buffer) to less than 20° after prewetting with only 5% PS20. For the hydrophilized Durapore PVDF membrane, the contact angle decreases from 46 ± 11° (buffer) to 9 ± 1° at 5% PS20. In contrast, the Supor PES membrane exhibits a small increase in contact angle from 7.0 ± 0.1° to 13 ± 1° on prewetting with 5% PS20, which may be due to a different orientation of the PS20 surfactant on the surface of this very hydrophilic polymer. The contact angle for the Isopore PCTE membrane falls from ∼80° (buffer) to roughly 40°–45° at 5% PS20 and continues to decrease at higher PS20 concentrations, reaching values in the ∼25° range by 20% PS20.

These variations in contact angle arise from differences in base‐polymer chemistry (PVDF, PES, and PCTE) and surface treatments, such as the acrylate coating on the hydrophilic Durapore membrane [21]. The PVDF filters appear to interact more strongly with the nonionic PS20 surfactant compared to PES or PCTE, leading to sharper reductions in contact angle. As the PS20 concentration increases, the contact angles of all filters tend to converge to values around 25°, suggesting that the contact angle under these conditions is determined primarily by the properties of the adsorbed/deposited layer of PS20 and not the surface characteristics of the underlying polymer.

### 3.3 Effect of PS20 on Filtration Behavior

**FIGURE 5:**
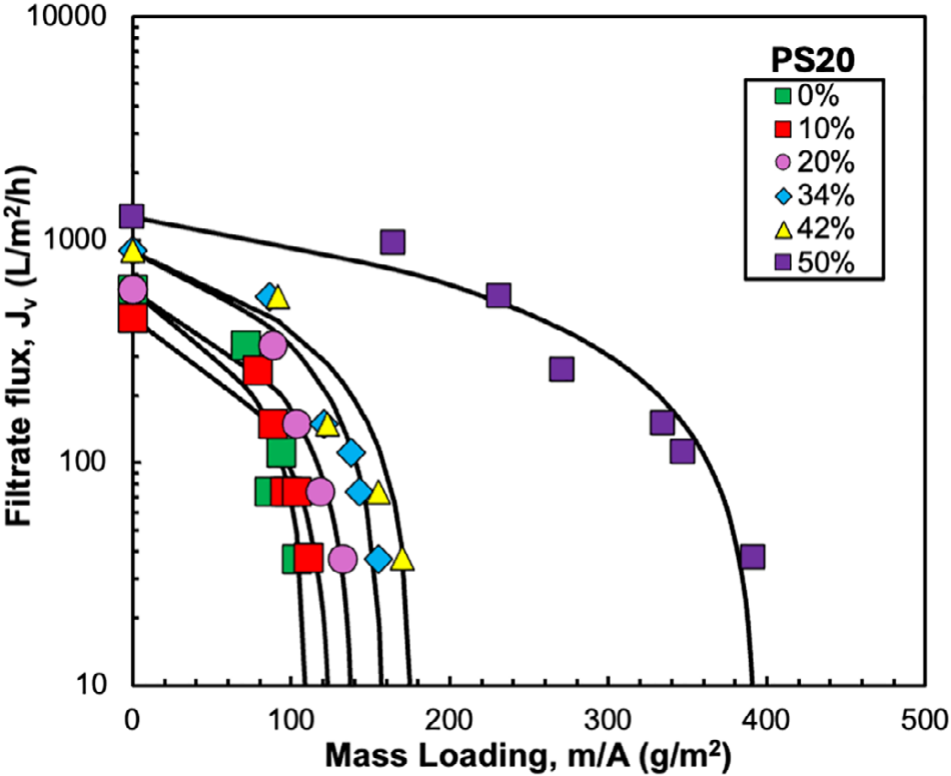
Filtrate flux profiles for constant pressure filtration of nanoemulsions through the Durapore hydrophilic filter prewetted with PS20 at varying concentrations at 280 kPa. Solid curves are model fits generated using the linearized form of the complete pore blockage model with the best fit values of the pore blockage parameters given in Table 2.

Filtrate flux data during constant pressure filtration of the NE through the hydrophilic Durapore membrane prewet with varying concentrations of PS20 are shown in Figure 5. The filtrate flux was evaluated by timed collection of permeate samples, with the volumetric flow rate normalized by the membrane area. The flux is plotted as a function of the loading, which is defined as the cumulative mass of NE processed per unit membrane area. The loading was calculated directly from the measured permeate volume by multiplying by the concentration of the feed, which was determined directly by the absorbance at 450 nm. The initial filtrate flux generally increases with increasing PS20 concentration, going from 590 L/m^2^/h for the hydrophilic Durapore prewet with just buffer (0% PS20) to 1260 L/m^2^/h for the filter prewet with 50% PS20.

The solid curves in Figure 5 are model fits developed using the complete pore blockage model as discussed by Kapila et al. [8]:

(2) $$J=J_{0}\left(1-\frac{k_{p}m}{A}\right)$$

where *J*_0_ is the initial flux (determined by extrapolation of the data back to zero throughput), *k*_*p*_ is the pore blockage parameter, determined by minimizing the sum of the squared residuals between the model and data, *m* is the mass of the filtered product, and *A* is the area of the filter. The filtrate flux data are concave down on the semi‐logarithmic plots, consistent with the behavior predicted by the classical pore blockage model. The model fits are in good agreement with the experimental data, allowing easy evaluation of the filter capacity, defined as the loading at which the filtrate flux decreases to 0, that is, when *m*/*A* = 1/*k*_*p*_. The filter capacity increases with increasing PS20 concentration in the prewetting solution, rising from 110 g/m^2^ for filters prewet with buffer alone (0% PS20) to 390 g/m^2^ for filters prewet with a 50% PS20 solution.

The calculated values of the initial filtrate flux, *J*_o_, and the pore blockage parameter, *k_p_*, are summarized in Table 2. The initial filtrate flux was relatively independent of the PS20 concentration below 20% PS20 but then increased significantly as the PS20 concentration increased from 20% to 50%. Note that this increase in initial flux is exactly opposite to the reduction in buffer permeability upon prewetting with PS20; the buffer permeability of the Durapore hydrophilic PVDF filter decreased by 21% after prewetting with 50% PS20. Increasing the PS20 concentration from 0% to 50% resulted in a monotonic decrease in *k_p_*, indicating a progressive reduction in pore‐blocking propensity with higher surfactant content during prewetting. This is likely due to a change in interactions between the NE droplets and the membrane pores, with the droplets able to squeeze more easily through the pores of the membranes that were prewet with a higher PS20 concentration. Note that this behavior was not directly correlated with the change in contact angle, with data for the Durapore hydrophilic PVDF filter showing a slight increase in contact angle at very high PS20 concentrations. This may reflect differences in PS20 adsorption/deposition on the surface of the filter (which determines the contact angle) versus within the porous structure (which is likely dominant in determining the rate of pore blockage).

**TABLE 2:** Effect of PS20 concentration used for prewetting on the initial filtrate flux, pore blockage parameter, and the mean hydrodynamic diameter and polydispersity index of the collected permeate during NE filtration through the Durapore hydrophilic PVDF filter at a constant pressure of 280 kPa (40 psi).

PS20 concentration (% *v*/*v*)	Initial filtrate flux, *J*_o_ (L/m^2^/h)	Pore blockage parameter, *k_p_* (m^2^/g)	Mean hydrodynamic diameter (nm)	Polydispersity index, PDI
0	590	0.0089 ± 0.0008	180 ± 3	0.25 ± 0.04
10	440	0.0079 ± 0.0009	181 ± 3	0.26 ± 0.04
20	590	0.0071 ± 0.0007	—	—
34	890	0.0063 ± 0.0005	182 ± 3	0.27 ± 0.04
42	890	0.0056 ± 0.0009	182 ± 3	0.25 ± 0.04
50	1260	0.0025 ± 0.0004	179 ± 3	0.28 ± 0.04

The final two columns of Table 2 show the mean hydrodynamic diameter and the polydispersity index for the collected permeate after filtration of the NE through the membranes examined in Figure 5 as determined by DLS. The feed had a mean droplet size of 156 ± 6 nm and a polydispersity index of PDI = 0.41 ± 0.08. All of the permeate samples showed a larger mean hydrodynamic diameter of 180 ± 2 nm and a smaller PDI than that for the feed, with no statistical difference between the data obtained after filtration through the pristine Durapore membrane (prewet with just buffer) or with any of the membranes prewet with the different PS20 solutions. The reduction in PDI is likely due to the removal of some very large nanodroplets/aggregates [8]. The increase in mean droplet size is surprising; this may be due to fusion of some of the smaller nanodroplets during passage through the sterile filter. The NE yield was greater than 94% for all conditions based on UV absorbance measurements of the feed and permeate samples.

### 3.4 Behavior of Different Sterile Filters

**FIGURE 6:**
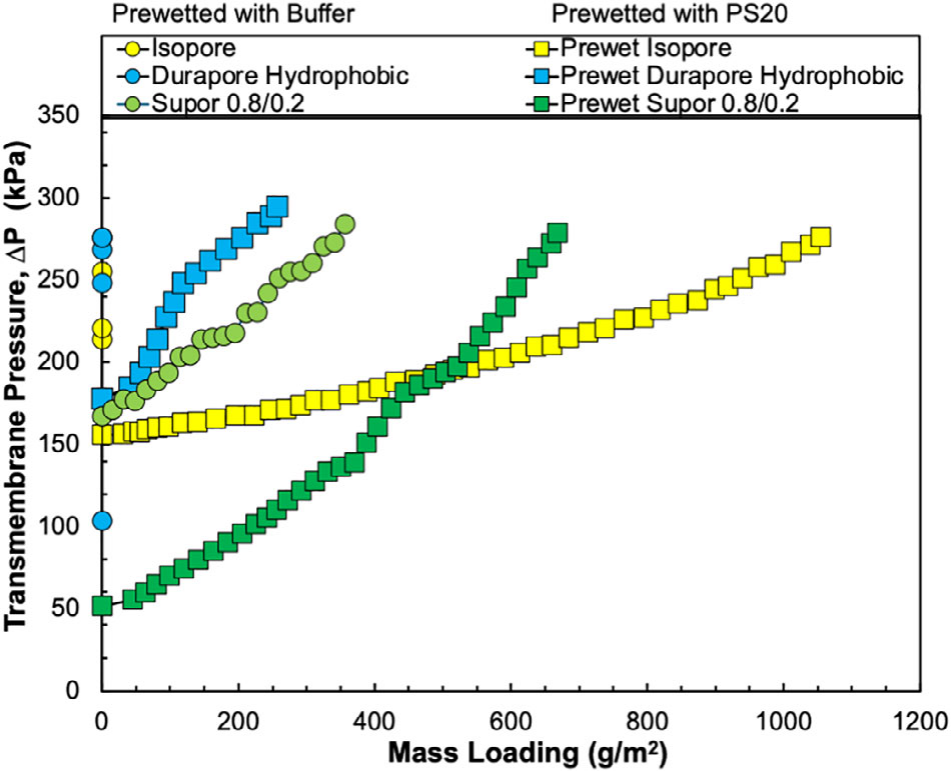
TMP during NE filtration through the Isopore, Durapore hydrophobic, and Supor 0.8/0.2 µm filters at a constant filtrate flux of 42 L/m^2^/h. Data are shown for membranes prewet with histidine buffer alone and with histidine buffer containing 20% PS20.

Given the widespread use of constant‐flux operation in industrial practice, additional NE filtration experiments were performed at a constant filtrate flux of 42 L/m^2^/h. Figure 6 shows results for the hydrophobic Durapore PVDF, polycarbonate Isopore, and Supor 0.8/0.2 µm PES filters, prewet with either histidine buffer (alone) or with histidine buffer containing 20% PS20. The Isopore and Durapore filters prewet with just histidine buffer have almost no capacity for the NE, with the TMP increasing to > 280 kPa (40 psi), which is the maximum pressure limit used in these experiments, within < 5 g/m^2^. In contrast, the membranes prewet with a solution containing 20% PS20 showed a much slower increase in TMP, with the prewet hydrophobic Durapore having a capacity of 260 g/m^2^ while the prewet Isopore has a capacity of more than 1000 g/m^2^. In both cases, there was no measurable flow until the TMP reached at least 150 kPa (22 psi), which is the pressure required to push the NE droplets through the small pores in the sterile filters. This is discussed in more detail in our previous publication [8]. Our hypothesis is that the dramatic increase in capacity for the hydrophobic Durapore and Isopore filters after prewetting with 20% PS20 is directly due to the increase in hydrophilicity (as discussed previously in Figure 4), which facilitates the passage of the oil nanodroplets (which are coated with surfactant) through the surfactant‐coated pores. A similar enhancement was observed with the Supor 0.8/0.2 µm sterile filter, although in this case the filter prewet with just buffer showed a capacity of nearly 350 g/m^2^, consistent with the very hydrophilic character of the base filter. Prewetting the Supor 0.8/0.2 µm filter with PS20 substantially reduced the initial TMP, from 175 kPa for the filter prewet with just histidine buffer to < 50 kPa when the filter was prewet with 20% PS20, with a corresponding 2‐fold increase in the filter capacity. A more detailed discussion of the effect of prewetting on the Supor 0.8/0.2 µm membrane is provided in the Supporting Information.

**FIGURE 7:**
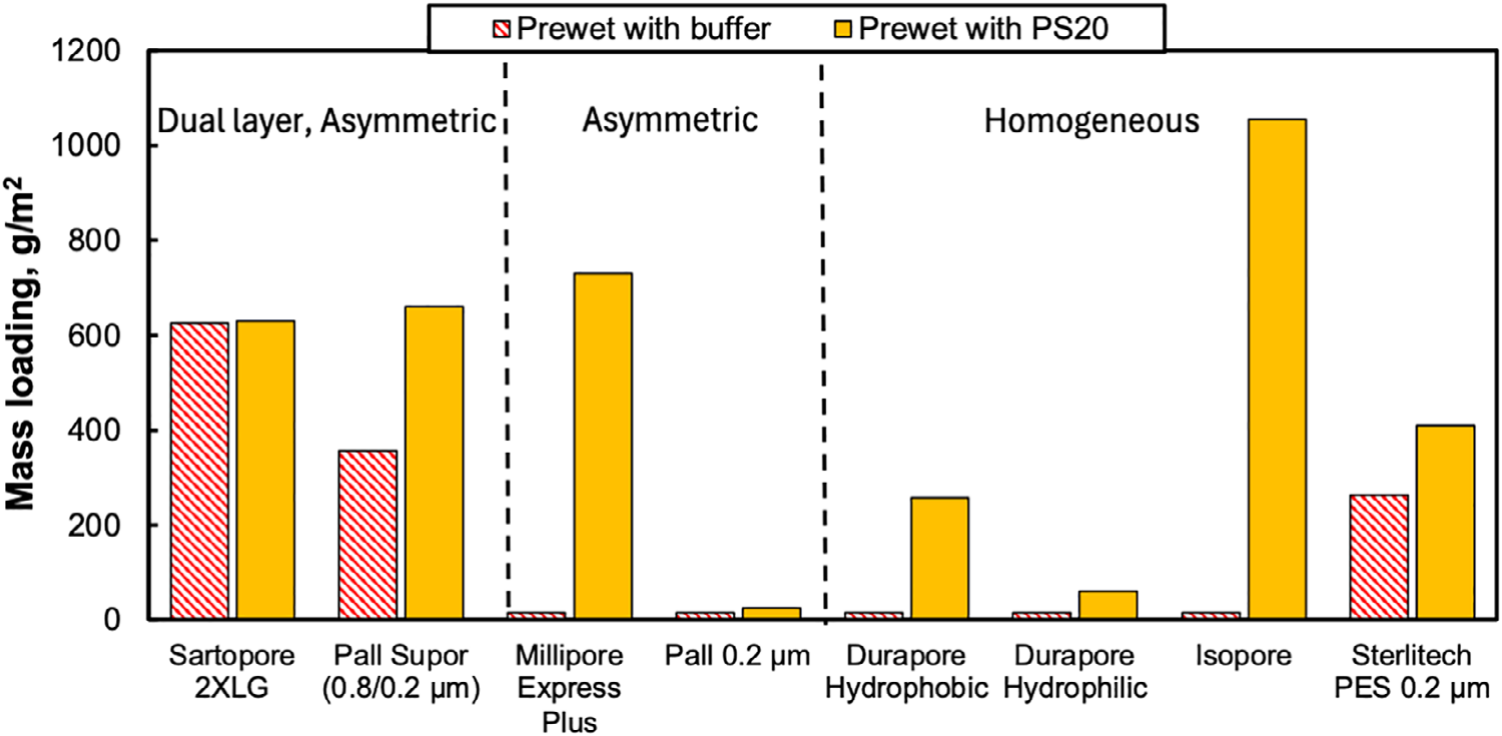
Capacities of the different 0.2 µm rated sterile filters during NE filtration at a constant flux of 42 L/m^2^/h for filters prewet with either buffer alone or buffer containing 20% PS20.

Figure 7 summarizes the results obtained with the full range of sterile filters, grouped based on the sterile filter morphology. The greatest capacity (more than 1000 g/m^2^) was obtained with the Isopore membrane that was prewet with PS20, even though this membrane had almost no capacity when prewet with just buffer. In contrast to the Supor 0.8/0.2 µm filter, in which the capacity increased to 350 g/m^2^ when prewet with 20% PS20, the 0.2 µm layer of this filter alone showed almost no capacity (< 20 g/m^2^) under either condition, demonstrating the critical importance of the prefilter layer in increasing the performance of the dual‐layer sterile filters. The Sartopore 2XLG dual layer filter showed essentially identical performance prewet with buffer alone and with buffer + PS20, while the hydrophilic Durapore and the Pall Supor 0.2 µm layer alone showed essentially no capacity under either condition. The capacity of the Durapore hydrophilic filter during constant flux operation was much less than that during constant pressure filtration (data in Figure 5); similar behavior was seen with the other filters. The capacity of the Millipore Express Plus increased by nearly 100‐fold after prewetting with PS20, going from <20 L/m^2^ to more than 700 L/m^2^. These changes in capacity were not directly correlated with the reduction in contact angle of the filters prewet with PS20, again suggesting that the fouling behavior is determined primarily by changes in pore structure/wettability within the depth of the porous structure and not simply on the external surface.

## 4 Conclusions

Obtaining high capacity during sterile filtration is critical to the successful commercialization of NE‐based drug products. Some commercial sterile filters, which show excellent capacity with protein therapeutics (e.g., monoclonal antibodies) showed almost no capacity with the NE. For example, the Millipore Express Plus and Durapore filters show a capacity of less than 20 g/m^2^ during constant flux filtration of the NE, while the Sartopore 2XLG had a capacity of more than 600 g/m^2^. The results presented in this paper demonstrate for the first time that it is possible to significantly enhance the performance of many of these filters by prewetting with PS20 in buffer. This effect was particularly dramatic for the Millipore Express Plus, which showed nearly a 100‐fold increase in capacity, with a similarly large increase seen for the Isopore polycarbonate track‐etch membrane. Even the Pall Supor 0.8/0.2 µm dual‐layer sterile filter showed nearly a doubling of the capacity after prewetting with 20% PS20. In contrast, the Sartopore 2XLG showed high capacity both with and without prewetting using PS20, suggesting that this sterile filter has both an appropriate pore size and surface chemistry for NE filtration.

The change in surface properties of the different membranes was confirmed by both FTIR and contact angle measurements and was also seen in SEM images of the Durapore hydrophilic filter before and after prewetting with PS20. The reduction in contact angle was typically greatest for the more hydrophobic materials (e.g., the Durapore hydrophobic filter and the Isopore polycarbonate), with the contact angle for a wide range of filter materials attaining a value around 25° after prewetting with solutions containing very high concentrations of PS20.

Constant pressure filtration experiments with the Durapore hydrophilic PVDF filter showed an increase in initial filtrate flux with increasing PS20 concentration even though the filters prewet with PS20 had a lower buffer permeability. This unusual behavior is a direct result of the unique filtration characteristics of the NE, which requires that the nanodroplets deform to enter and pass through the narrow pores. In this case, prewetting with PS20 facilitates passage of the NE droplets, likely by reducing frictional interactions between the surfactant‐coated surfaces of the droplets and the pores. Prewetting also reduced the rate of pore blockage, which in turn significantly increased the capacity of many of these filters. Future studies will be required to demonstrate the generalizability of these results to other NE formulations made with different surfactants.

## Author Contributions

**Shreya Kapila**: data curation, formal analysis, investigation, and writing – original draft. **Randal J. Soukup**: funding acquisition, conceptualization, methodology, resources, and writing – review and editing. **Marissa E. Bradley**: methodology, resources, and writing – review and editing. **David Boyd**: methodology, resources, and writing – review and editing. **Andrew L. Zydney**: conceptualization, funding, supervision, and writing – review and editing.

## Funding

This work was funded by Merck Sharp & Dohme LLC, a subsidiary of Merck & Co., Inc., Rahway, NJ, USA.

## Conflicts of Interest

Randal J. Soukup, Marissa E. Bradley, and David Boyd are employees of Merck & Co., which has interest in the use of nanoemulsions for their drug products. Andrew Zydney is a consultant for Merck & Co.

## Supporting information




**Supporting File**: biot70280‐sup‐0001‐SuppMat.docx.

## Data Availability

The data that support the findings of this study are available from the corresponding author upon reasonable request.
