# Supplementary material for: Modulating Membrane Surface Properties via Prewetting With Polysorbate 20 to Improve Sterile Filtration of Nanoemulsions
Source: Biotechnol J. 2026 Jul 9;21(7):e70280. doi: 10.1002/biot.70280 (PMC13347756; doi:10.1002/biot.70280)
Supplement: Supplementary file 1 — Supporting File: biot70280‐sup‐0001‐SuppMat.docx. [file BIOT-21-e70280-s001.docx]

**Modulating membrane surface properties via prewetting with polysorbate 20 to improve sterile filtration of nanoemulsions**

**Supplementary information**

Figure S1 shows the effects of prewetting the Pall Supor 0.8/0.2 µm sterile filter with varying concentrations of PS20 on the pressure profiles during NE filtration at a constant filtrate flux of 42 L/m^2^/h. Prewetting led to a significant reduction in the initial TMP, which decreased from 175 kPa for the filter prewet with just histidine buffer to <70 kPa when the filter was prewet with the different PS20 concentrations.

Prewetting the Pall Supor 0.8 / 0.2 µm sterile filter with PS20 also provided a significant increase in filter capacity from 350 g/m² for the filter that was simply prewet with the histidine buffer to 500 g/m² for the filter prewet with 10% PS20 and 700 g/m² after prewetting with 50% PS20. This increase in capacity appeared to be due to a combination of the lower initial TMP and the lower rate of fouling (as measured by the TMP gradient) at the start of the filtration. For example, the TMP gradient for the filter that was prewet with just buffer was nearly constant at a value of 0.32 kPa m^2^/g throughout the filtration. In contrast, the filter that was prewet with 50% PS20 shows an initial TMP gradient of only 0.19 kPa m^2^/g, but this increased during the filtration to a value of 0.44 kPa m^2^/g beyond 400 g/m^2^. The physical basis for this change in fouling behavior is unclear.


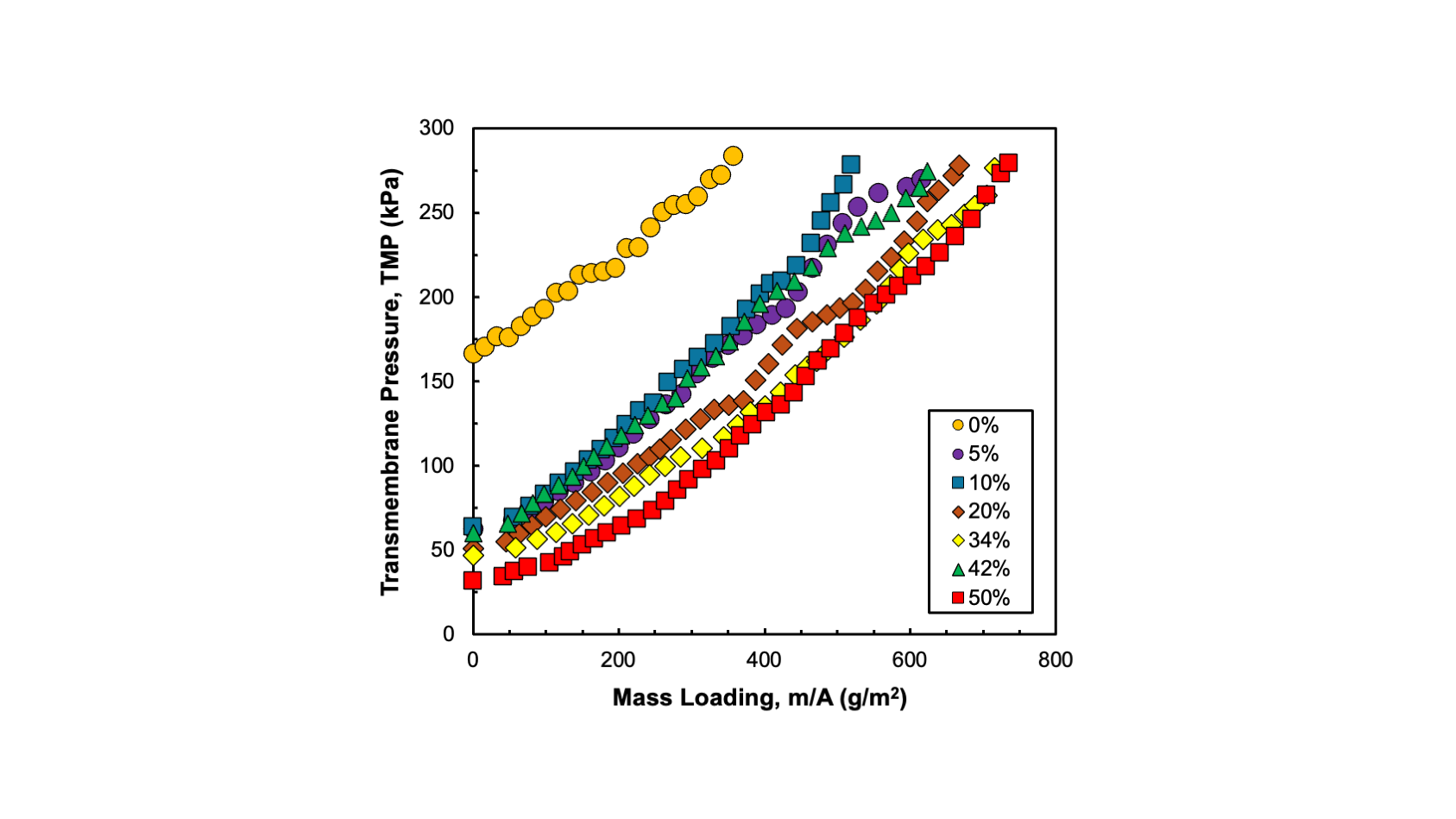


Figure S1: TMP across the Supor 0.8/0.2 µm sterile filter during NE filtration at a constant flux of 42 L/m^2^/h for filters pre-wet with different concentrations of PS20.

Table S1 summarizes the effects of prewetting the hydrophilic Durapore membrane with different PS20 concentrations on both the surface properties and filtration performance. The contact angle decreased sharply, from 46 ± 10^o^ for the membrane prewet with just the histidine buffer to 8.4 ± 1^o^ for the membrane prewet with 10% PS20. Prewetting with 10% PS20 also caused a very large drop in the membrane hydraulic permeability; there were no statistical differences in the permeability between the filters prewet with the different PS20 concentrations. In contrast, the membrane capacity and initial filtrate flux both increase significantly when the hydrophilic Durapore membrane was prewet with solutions containing higher PS20 concentrations. These results show the complex effects of PS20 on the sterile filter and its performance during NE filtration.

**Table S1** – Effect of PS20 concentration on the contact angle, hydraulic permeability, and filtration characteristics of the hydrophilic Durapore membrane.

| **% PS20 used for prewetting** | **Contact Angle (°)** | **Hydraulic Permeability (Lm^-2^h^-1^kPa^-1^)** | **Capacity (g/m^2^)** | **Initial Filtrate Flux (LMH)** | **Pore blockage parameter, k_block_ (m^2^/g)** |
| --- | --- | --- | --- | --- | --- |
| 0 | 46 ± 10 | 60 ± 3 | 100 | 590 | 0.009 |
| 10 | 8.4 ± 1 | 41 ± 7 | 106 | 445 | 0.008 |
| 20 | 9.0 ± 0.1 | 52 ± 4 | 130 | 595 | 0.007 |
| 34 | 7.0 ± 0.4 | 46 ± 6 | 140 | 890 | 0.006 |
| 50 | 19 ± 5 | 46 ± 10 | 345 | 1257 | 0.0025 |
